# Antimalarial activity of ulein and proof of its action on the *Plasmodium falciparum* digestive vacuole

**DOI:** 10.1186/1475-2875-9-S2-O9

**Published:** 2010-10-20

**Authors:** Alaíde Braga de Oliveira, Maria Fâni Dolabela, Marinete Marins Póvoa, Cid Aimbiré M Santos, Fernando de Pilla Varotti

**Affiliations:** 1 Universidade Federal de Minas Gerais, Av. Alfredo Balena, 190 - Campus Saúde, 30130-100 Belo Horizonte, Brazil; 2 Universidade Federal do Pará, Rua Augusto Correa,01 - Guama 66075-110 Belém, Brazil; 3 Instituto Evandro Chagas, Avenida Almirante Barroso, 492 - Marco 66093-971, Belém, Brazil; 4 Universidade Federa do Paraná, Rua General Carneiro, 181 - Alto da Glória, 80060-150, Curitiba, Brazil; 5 Universidade Federal de São João Del Rei, Praça Frei Orlando, 170, Centro, São João del-Rei, Minas Gerais 36307-352, Divinópolis, Brazil

## Background

For thousands of years, plants have formed the basis of traditional medicine systems. The first drug to be used against malaria was quinine, which was isolated from South American *Cinchona* spp., in 1820. More recently, artemisinin, isolated in China from *Artemisia annua* L. and derivatives have been used successfully against malaria that has become resistant to chloroquine. We have been investigating plants traditionally used in Brazil to treat malaria and fevers.

## Materials and methods

Screening of plant extracts for inhibition of *Plasmodium falciparum* growth was assayed by the [^3^H]-hypoxanthine methodology. EtOH extract from *Aspidosperma parvifolium* was shown to be active (W2: IC_50_ = 42.51 ± 6.33 μg/ml) and has afforded the alkaloid ulein, which has shown a good antiplasmodial activity (W2: IC_50_ = 0.98 ± 0.20 μg/ml). Possible targets for ulein have been investigated by confocal microscopy using a proton fluorescent probe (BCECF-AM) in *P. falciparum* synchronous culture of W2-infected red blood cells by comparison with mefloquine (MQ) and bafilomycin A1 (BAF). Dynamic imaging was performed with the LSM 510 laser-scanning microscope (Carl Zeiss), using LSM 510 software (version 2.5), in the Axiovert 100 M microscope equipped with a 63xoil immersion objective. Software-based analysis of data allowed fluorescence imaging in a selected cell as a function of time.

## Results

Dynamic images have shown that ulein (5 ng/ml), was able to mobilize protons altering the pH gradient in the digestive vacuole (DV), like MQ, a weak-base antimalarial quinoline. However, after the addition of ulein, BAF (4 μM), an inhibitor of the H+ pump from acidic compartments of eukaryotic cells, had no action on the DV suggesting that it is a target for ulein action.

## Conclusions

This work shows that ulein is able to act on the DV, probably due to its alkaloid structure. Whether there is a participation of PfCRT, PfMDR1, and PfATPase6 in ulein action is still an open question. Its action on calcium homeostasis needs to be further investigated. These data disclose ulein as a potential antimalarial drug.

